# Significant Differences in the Gut Bacterial Communities of Hooded Crane (*Grus monacha*) in Different Seasons at a Stopover Site on the Flyway

**DOI:** 10.3390/ani10040701

**Published:** 2020-04-17

**Authors:** Fengling Zhang, Xingjia Xiang, Yuanqiu Dong, Shaofei Yan, Yunwei Song, Lizhi Zhou

**Affiliations:** 1 School of Resources and Environmental Engineering, Anhui University, Hefei 230601, China; 2 Anhui Province Key Laboratory of Wetland Ecosystem Protection and Restoration (Anhui University), Hefei 230601, China; 3 Shengjin Lake National Nature Reserve of Anhui Province, Dongzhi 247200, China

**Keywords:** hooded crane, intestinal bacteria, alpha-diversity, seasonal fluctuation, migration, high-throughput sequencing

## Abstract

**Simple Summary:**

Intestinal bacterial taxa play an important role in maintaining the normal physiological ecology of animals. Many factors affect the composition and diversity of the intestinal bacterial community, including diet, environment and season. However, the unique life cycle of birds makes it interesting to study their gut bacteria, especially migratory birds. Birds use many habitats and food resources, which may influence their intestinal bacterial community structure during seasonal migration. Hooded crane (*Grus monacha*) is one such migrant waterbird that traverses long distances and occupies varied habitats. In this study, we investigated the diversity and differences in intestinal bacterial communities of hooded cranes over the migratory season. The intestinal bacterial alpha-diversity of hooded cranes in winter was significantly higher than fall and spring. The gut bacterial community composition differed significantly across the three seasons (ANOSIM, *P* = 0.001). This study provides baseline information on the seasonal dynamics of intestinal bacteria in migratory hooded cranes.

**Abstract:**

Intestinal bacterial communities form an integral component of the organism. Many factors influence gut bacterial community composition and diversity, including diet, environment and seasonality. During seasonal migration, birds use many habitats and food resources, which may influence their intestinal bacterial community structure. Hooded crane (*Grus monacha*) is a migrant waterbird that traverses long distances and occupies varied habitats. In this study, we investigated the diversity and differences in intestinal bacterial communities of hooded cranes over the migratory seasons. Fecal samples from hooded cranes were collected at a stopover site in two seasons (spring and fall) in Lindian, China, and at a wintering ground in Shengjin Lake, China. We analyzed bacterial communities from the fecal samples using high throughput sequencing (Illumina Mi-seq). Firmicutes, Proteobacteria, Tenericutes, Cyanobacteria, and Actinobacteria were the dominant phyla across all samples. The intestinal bacterial alpha-diversity of hooded cranes in winter was significantly higher than in fall and spring. The bacterial community composition significantly differed across the three seasons (ANOSIM, *P* = 0.001), suggesting that seasonal fluctuations may regulate the gut bacterial community composition of migratory birds. This study provides baseline information on the seasonal dynamics of intestinal bacterial community structure in migratory hooded cranes.

## 1. Introduction

Gut bacterial taxa play an important role in organismal health, including regulating digestion [1], nutrient uptake [2], fat metabolism [3], and vitamin synthesis [4]. A range of factors influence gut bacterial community composition and diversity, including diet [5], environment [6,7], and seasons [8]. However, the unique life cycle of birds, especially migratory birds, makes it interesting to study their gut bacterial community [9].

Migrant birds have a unique annual life cycle that involves seasonal migration and complex dietary habits [10]. Most migrant birds have stopover sites between their breeding and wintering grounds. Birds have high energy demands during seasonal migration [11] and must rest at stopover sites, particularly when energy reserves are low, to feed and store fat for subsequent migratory flights [9]. Like other vertebrates, the guts of birds harbor a large number of bacterial taxa. Current studies suggest that the dominant gut bacterial phyla in birds are Firmicutes, Proteobacteria, Actinobacteria and Bacteroidetes [12]. These bacterial phyla maintain the normal physiological functions of birds during non-migratory, as well as migratory, periods [7]. Therefore, studying the gut bacterial community composition in migratory birds at their stopover sites is important for understanding their life history. The rapid development of molecular biotechnology provides researchers with more information about bird biology during migration.

The hooded crane (*Grus monacha*) is a vulnerable (VU) species listed on the International Union for Conservation of Nature (IUCN) Red List, with an estimated global population of ~11,600 [13], which breeds in a range of areas across Russia and Asia, and winters mainly in Southern Japan, Southern Korea, and the Middle and Lower Yangtze River floodplain in China [13,14]. The cranes have three key stopover sites during spring and fall migration, including the region surrounding Muraviovka Park in Russia, the Songnen Plain in Northeast China, and the west coast of South Korea. They spend a relatively long time at wintering and breeding grounds (about 5 months), but a relatively short time (~2 weeks) at the stopover sites on the flyway [15]. The winter population in China consists of 1050~1150 individuals, with ~10,500 wintering in Japan [13].

Hooded cranes use diverse habitats on their flyway. Lindian is a key stopover site in the Songnen Plain, Daqing City, Heilongjiang Province, China, due to its abundant food resources including corn, rice, and soybeans [16,17]. Nearly a decade of research has shown that over 4000 individuals (34% of the global population) occupy this region during each migration [16]. Shengjin Lake is a shallow lake in the middle and lower Yangtze River floodplain [18], and one of the largest wintering grounds in the world. The main food resources for hooded cranes are *Vallisneria natans*, *Potamogeton malaianu*, and rice grains [19]. In addition, the abundance and availability of food can influence the foraging strategies of the hooded crane, which may affect their intestinal physiology and ecology [20]. We hypothesized that: (a) as different habitats provide hooded cranes with different food resources, the diversity and the composition of their intestinal bacterial communities differ between stopover and wintering sites, and; (b) different seasons may offer hooded cranes with different food abundance at the same site, thus differences in the diversity and composition of the intestinal bacterial community between the spring and fall stopover sites may exist. In this study, we evaluated the intestinal bacterial communities of hooded cranes by using 16s rRNA high-throughput sequencing during the fall, winter, and spring.

## 2. Materials and Methods 

### 2.1. Ethics

To avoid disturbing the animals, we collected fecal samples after foraging. Permissions were obtained from the Anhui Shengjin Lake National Nature Reserve and Lindian County Wetland Management Bureau.

### 2.2. Sample Collection and Study Sites

Samples were collected at Shengjin Lake (30.25~30.50°N, 116.92~117.25°E) (Figure S1A), an important wintering ground, and the other in Lindian (46.73~47.48°N, 124.3~125.35°E) (Figure S1B), Songnen Plain in northeast China, a key stopover site during the fall and spring [15]. During the spring and fall migration periods, 20 samples of hooded crane feces were collected each on October 16, 2017 (47.13°N, 124.57°E) and March 26, 2018 (47.21°N, 124.58°E) at western Lindian, respectively. In the winter period, 20 samples were collected on January 19, 2018 at Shengjin Lake (30.34°N, 117.01°E).

Prior to sample collection, hooded cranes were monitored to visualize areas that had been foraged by ≥50 birds. We selected areas in which no other bird foraging had been observed in a 50 m radius. To reduce the likelihood of any repetition in terms of sampling, interval distances were set as ≥5 meters. Samples were collected into sterile tubes when the cranes vacated the sampling site and immediately stored on ice prior to assessment [21]. All samples were collected with disposable gloves to avoid contamination across samples. Samples were thawed only once prior to analysis.

### 2.3. Fecal DNA Extraction

DNA extraction was performed using commercially available Fast DNA^TM^ Spin Kits for Soil samples (MP Biomedicals, Irvine, CA, USA) and quantified on a NanoDrop ND-1000 (Thermo Fisher Scientific, Wilmington, DE, USA). The extracted DNA was stored in a freezer prior to analysis.

### 2.4. Species Identification

BirdF1(TTCTCCAACCACAAAGACATTGGCAC)-BirdR1(ACGTGGGAGATAATTCCAAATCCTG) were used as standard primers for amplification of the COI barcoding region to confirm the species of each bird [22]. Polymerase chain reactions (PCRs) were performed using SuperMix (2 × EasyTaq® PCR SuperMix (+dye), TRANSGEN, Beijing, China). PCR parameters: 5 min at 95 °C, 30 s at 95 °C for 35 cycles, 45 s at 55 °C, 90 s at 72 °C, and 10 min at 72 °C for final extension. Final PCR products were subjected to DNA sequencing and blast searched (>97% identity) on the NCBI database. Samples identified as belonging to hooded crane were subjected to high-throughput sequencing (*n* = 60 samples in this study).

### 2.5. Amplicon Libraries

Purified DNA samples (~50 ng) were subject to PCR using primers (F515/R907) to amplify the V4-V5 hypervariable regions of 16S rRNA for the analysis of the gut bacteria [23]. PCRs consisted of: dNTPs (200 μM), forward (F515: GTGCCAGCMGCCGCGG) and reverse primers (R907: CCGTCAATTCMTTTRAGTTT) (0.4 μM), and 2 U of Taq DNA polymerase (TaKaRa, Kyoto, Japan). Reaction conditions were as follows: 94 °C for 5 min, 94 °C for 45 s × 35 cycles, 55 °C for 45 s, 45 s at 72 °C, and 10 min final extension at 72 °C. PCRs were performed in triplicate on two independent occasions. Negative and positive controls were assessed to ensure accuracy [6]. Mis-tagging was controlled using blank tag combinations [24]. PCR products were pooled and gel purified to a final concentration of 10 pg. Library preparation and pyrosequencing were performed at Majorbio (Shanghai, China) with the Illumina Mi-Seq platform (PE 300).

### 2.6. Data Analysis 

QIIME (V.1.9) was used for all data analysis [25]. Sequences ≤250 bp with quality scores of 30 were discarded. Included sequences were clustered according to Operational Taxonomic Units (OTUs; ~97% similarity) using UCLUST [26]. Chimeras and singleton OTUs were removed. The most abundant sequence in each OTU was selected as the representative sequence, and the representative sequences were then taxonomically classified into phylum, class, order, family, genus, and species via the Ribosomal Database Program (RDP) classifier (UNITE v.8.0) [27]. Sequences were then aligned using PyNAST [25]. To ensure equal rarefication amongst the samples, 26,000 subset sequences were randomly collected (repetition ≥20 for each sample) for the comparison of bacteria and community diversity. Alpha-diversity indices (including Shannon and Chao1 assessments) were calculated from rarefied samples to reveal both diversity and species richness. The raw data obtained in this study have been submitted to the NCBI Sequence Read Archive (accession number SRP226548).

### 2.7. Statistics

Non-metric multidimensional scaling (NMDS) was used to assess beta-diversity. Groups were compared across seasons using a one-way analysis of similarity with permutations of 999 [28] using the vegan package in R software (V.3.1.0). The diversity in OTUs across each sampling season was compared using SIMPER analysis [29]. The labdsv package was used for indicator analysis. Linear discriminant analysis (LDA) effect sizes (LEfSe) were assessed to rank the most abundant modules in each season using a parametric Kruskal-Wallis rank-sum test (Alpha-value: 0.05; effect size threshold: 2) for biomarker identification [30]. LEfSe was performed using the Galaxy workflow and diversities are shown as the mean ± SD. The data was analyzed using the Kolmogorov–Smirnov test. One-way ANOVA was used to analyze alpha-diversity indices (Chao1, OTU richness, PD, Shannon) and relative abundance of dominant bacteria (>1%) across samples (for the normally distributed data, *p* > 0.05) or Mann–Whitney–Wilcoxon test (for non-normally distributed data, *p* < 0.05). *p* values < 0.05 were deemed significant; *p* values < 0.01 indicated a high level of significance (Table S1).

## 3. Results

### 3.1. Intestinal Bacterial Alpha-Diversity of the Hooded Crane

**Figure 1:**
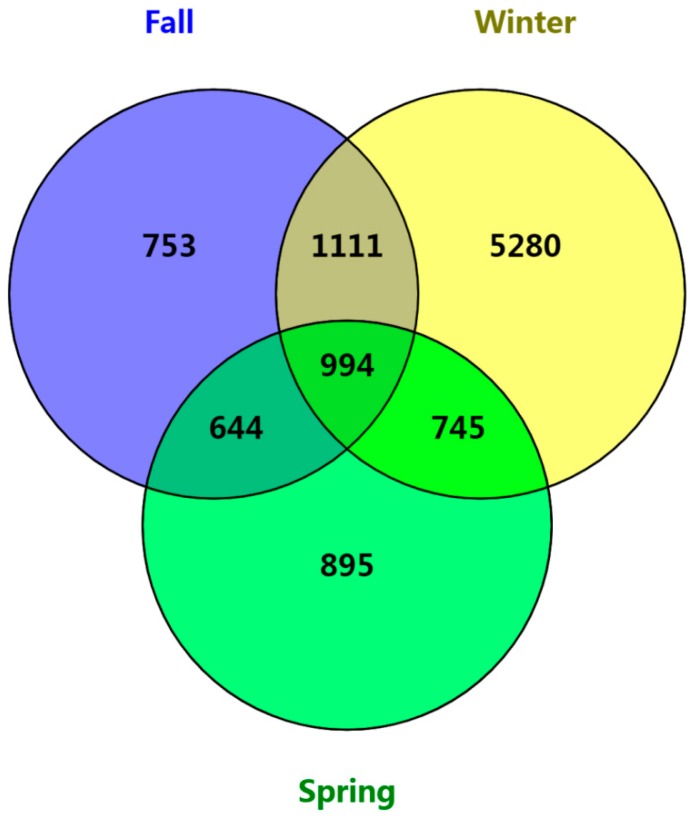
Venn diagram showing the unique and shared intestinal bacterial operational taxonomic units (OTUs) of hooded crane among the three seasons.

We acquired 2,220,412 high-quality bacterial sequences using prime set F515/R907, ranging from 26,799 to 59,237 sequences per sample (mean = 37,007). In total, 10,422 OTUs were obtained (ranging from 156 to 2377; 97% similarity), 9.5% of which (994) were identified in all three seasons. During fall and winter, the number of shared bacterial OTUs was 1111 (10.7%). During winter and spring, the number of shared bacterial OTUs was 745 (7.1%). During fall and spring, the number of shared bacterial OTUs was 644 (6.2%). The number of unique gut bacterial OTUs were 753 (7.2%), 5280 (50.7%) and 895 (8.6%) in fall, winter and spring, respectively (Table S2, Figure 1).

**Figure 2:**
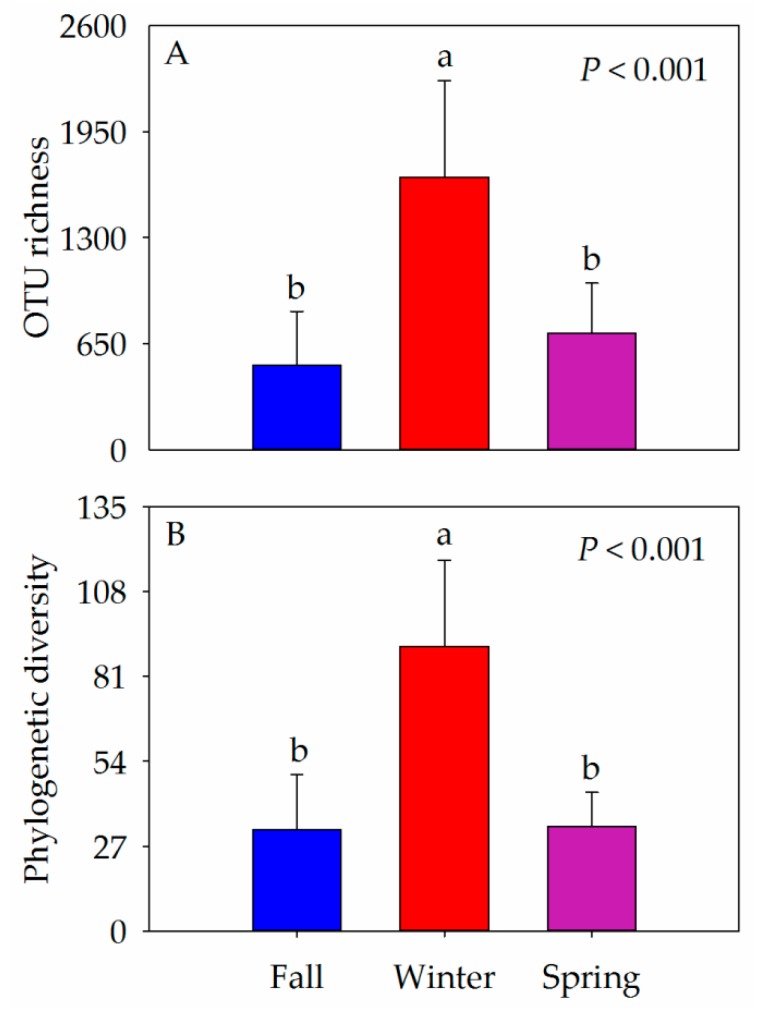
Gut bacterial alpha-diversity (A: OTU richness; B: Phylogenetic diversity) of different seasons. Different letters represent significant differences from one-way ANOVA by Tukey’s HSD comparisons (*P* < 0.05). Bars represent mean; error bars indicate standard deviation.

The OTU richness and phylogenetic diversity were used to evaluate gut bacterial alpha-diversity of hooded cranes across the three seasons. The bacterial alpha-diversity of hooded crane was significantly higher in winter relative to the other seasons (i.e., fall and spring; Figure 2). 

### 3.2. Intestinal Bacterial Community Composition

**Figure 3:**
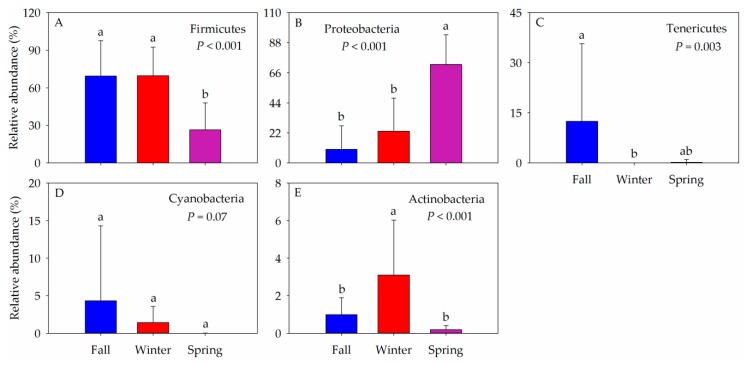
Relative abundance of the dominant bacterial phyla in three seasons. (**A**) Firmicutes, (**B**) Proteobacteria, (**C**) Tenericutes, (**D**) Cyanobacteria, and (**E**) Actinobacteria. Different letters represent significant differences in Tukey’s HSD comparisons (*P* < 0.05). Bars represent mean; error bars indicate standard deviation.

Firmicutes (55.19%), Proteobacteria (35.14%), Tenericutes (4.23%), Cyanobacteria (1.93%), and Actinobacteria (1.43%) were the dominant phyla across all samples (Figure 3, Table S3). The dominant intestinal bacterial classes were Bacilli (53.36%), Gammaproteobacteria (26.75%), Alphaproteobacteria (7.80%), Mollicutes (4.23%), Clostridia (1.81%), and Actinobacteria (1.23%) across all samples (Table S4). However, in different seasons, the proportion of the dominant intestinal bacterial phyla of the hooded crane was different (Figure S2, Table S3). The relative abundance of Firmicutes was higher in the fall compared to winter and spring (*p* < 0.001), while the relative abundance of Proteobacteria was the highest in the spring, and the lowest in the fall (*p* < 0.001). The relative abundance of Tenericutes was significantly higher in the fall than winter and spring (*p* = 0.006). The relative abundance of Actinobacteria was more abundant in the winter relative to other seasons (*p* < 0.001). Little difference in the relative abundance of Cyanobacteria was observed across the seasons (*p* = 0.07) (Figure 3).

**Figure 4:**
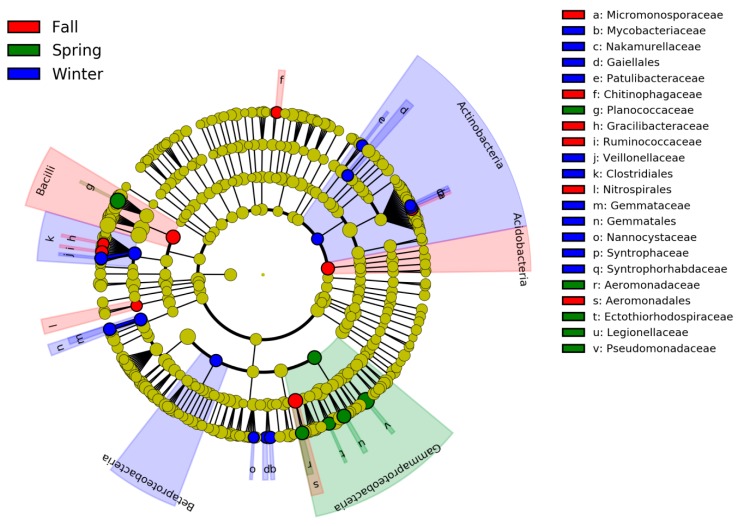
LEfSe analysis of the hooded crane gut bacterial biomarkers among three seasons. Cladogram represents the taxonomic hierarchical structure of the phylotype biomarkers identified among seasons. Identified phylotype biomarkers ranked by effect size (LDA > 2, *p* < 0.05). Red, phylotypes statistically overrepresented in fall; green, phylotypes statistically overrepresented in spring; blue, phylotypes statistically overrepresented in winter.

**Figure 5:**
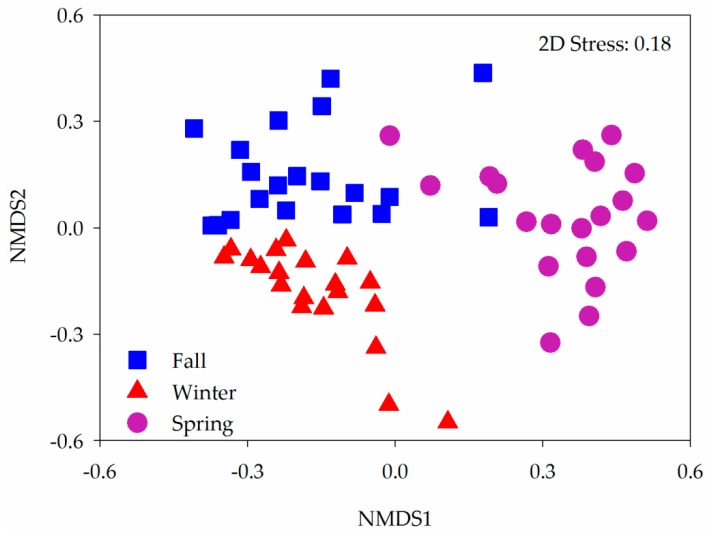
Non-metric multidimensional scaling (NMDS) showing intestinal bacterial community compositions of hooded cranes across three seasons.

Specific intestinal bacterial taxa in the cranes that differed across the three periods were identified by LEfSe. Two orders (Nitrospirales, Aeromonadales) and four families (Micromonosporaceae, Chitinophagaceae, Gracilibacteraceae, and Ruminococcaceae) were significantly more abundant in fall. Three orders (Gaiellales, Clostridiales, and Gemmatales) and eight families (Mycobacteriaceae, Nakamurellaceae, Patulibacteraceae, Veillonellaceae, Gemmataceae, Nannocystaceae, Syntrophaceae, and Syntrophorhabdaceae) were significantly more abundant in winter. Five families (Planococcaceae, Aeromonadaceae, Ectothiorhodospiraceae, Legionellaceae, and Pseudomonadaceae) were more abundant in spring (Figure 4). Bacterial community compositions of the hooded cranes were significantly different in the three periods (Figure 5). ANOSIM analysis confirmed the impact of the seasons on the bacterial community composition of the hooded cranes (*P* = 0.001) (Table 1). Although there is little difference in intestinal bacterial alpha-diversity of hooded cranes between the spring and fall at the stopover site (i.e., Lindian), the bacterial community composition is significantly different between them. SIMPER analysis was performed to identify the bacterial taxa primarily contributing to community dissimilarities between seasons. The results revealed that OTU_11063 (*Lactobacillus*; 15.80%), OTU_11375 (*Enterococcus*; 12.93%), OTU_11271 (Alphaproteobacteria; 10.60%), OTU_11646 (Mollicutes; 8.19%), and OTU_9558 (*Paenibacillus*; 3.43%) had a key influence on the community differences between the fall and winter. OTU_11063 (*Lactobacillus*; 13.29%), OTU_11375 (*Enterococcus*; 10.80%), OTU_11646 (Mollicutes; 7.02%), OTU_5995 (Enterobacteriaceae; 14.23%), and OTU_1494 (*Pseudomonas*; 5.87%) were the major OTUs responsible for differences between fall and spring. OTU_11063 (*Lactobacillus*; 15.61%), OTU_11271 (Alphaproteobacteria; 9.26%), OTU_5995 (Enterobacteriaceae; 13.60%), OTU_1494 (*Pseudomonas*; 5.52%), and OTU_6855 (Enterobacteriaceae; 5.42%) led to primary differences between winter and spring (Table 2). Indicator analysis was used to identify bacterial OTUs associated with the seasons. We identified 12, 21, and 21 indicator species in the fall, winter, and spring, respectively (Table S5).

## 4. Discussion

Migrant birds have a unique annual life cycle that involves seasonal migration and complex dietary habits. They have stopover sites between their breeding and wintering grounds with high energy demands during seasonal migration [11]. Like other vertebrates, the guts of birds harbor a large range of bacterial taxa. In this study, we found that the proportion of shared bacterial OTUs was very low among different seasons. Furthermore, we examined and compared the bacterial community composition and diversity of hooded crane across three seasons. The results showed that the bacterial community composition significantly differed across fall, winter and spring. In addition, the gut dominant phyla in hooded crane differed from those in mammals and reptiles [31,32]), while showing similarities to shorebirds [33], kakapo (*Strigops habroptilus*) [12], and other passerines [34,35]. 

Firmicutes can help hosts degrade non-digestible carbohydrates (i.e., dietary fiber) [21,36]. Thus, the enrichment of Firmicutes in guts of hooded cranes may contribute to energy intake and nutrient absorption [37]. Firmicutes was primarily consisted of *Lactobacillus ruminis* at the species level. *Lactobacillus ruminis* was closely related to the carbohydrate-active enzymes of beta-glucosidase, mannosidase, trehalose phosphorylase, fucosidase, and sialidase [38]. Significant differences in the distribution of Proteobacteria was also found, particularly for Gammaproteobacteria (26.75%) and Alphaproteobacteria (7.80%) in guts of hooded cranes. The relative abundance of Proteobacteria was positively associated with digestible carbohydrate [38,39]. Gammaproteobacteria was mainly composed of Enterobacteriaceae and Pseudomonadaceae, which was consistent with other studies found in guts of black-legged kittiwakes [40], Procellariiformes seabirds [41] and Canada geese [42]. Members of these families show cellulase activity and degrade aromatic compounds [43]. Thus, the results suggested that the enrichment of these families facilitates cellulose degradation and nutrient absorption for their hosts [37,44]. 

The intestinal bacterial community composition and diversity of hooded cranes showed significant differences between habitats. At Shengjin lake, the hooded cranes in their wintering habitat mainly feed on *Vallisneria natans*, *Potamogeton malaianus*, and rice grains [14]. However, in stopover site of Lindian, corn is the main food resource of hooded cranes [16,17]. It is known that food abundance and availability can influence the foraging strategies of hooded cranes, which may affect the physiology and ecology of their intestines [20]. Thus, the results suggested that variation in food resources and abundance may led to significant differences of bacterial community composition between the habitats. However, a significant difference in gut bacterial community composition and diversity was found between fall and spring migratory periods in the same region. Lindian grows a single crop per year and corn ripens in the fall. Thus, the hooded cranes might have relatively higher food resource availability in fall compared to the spring. Food abundance dictates foraging efficiency and energy production [16,20], which might be a crucial factor influencing the foraging strategies of migratory birds. Dramatic shifts in bacterial community composition of hooded cranes might be associated with the different food abundances between the two periods. 

Studies have shown that host physical activity can affect the gut bacterial community composition and diversity in rats [45]. Exercise and gut atrophy experienced during migration are also likely to influence the results found in two seasons at the stopover site [9]. Individual differences may also be factors affecting the gut bacterial community composition of hosts [40]. The cranes lived in breeding grounds for a long time period prior to arriving at Lindian in fall, while they lived in wintering grounds for extended time periods prior to arriving at Lindian in spring. Thus, they may have been exposed to different environments and food abundance prior to arriving at the stopover site [46,47]. So, we suspect that the shifts in bacterial community composition of the cranes between fall and spring may also arise from the variable diets and habitats that occur during migration several days prior to sampling [31,48]. 

## 5. Conclusions

The intestinal bacterial communities of the hooded cranes significantly differed among the three seasonal periods, suggesting that seasonal fluctuations might shape the gut bacterial community of migrant birds. Changes in food resources and/or abundances might lead to shifts in the gut bacterial community of the hooded cranes among seasons. Our results build a more complete picture of gut bacterial communities of migrant birds among different seasons. However, there were certain limitations of this study. Only 60 samples were assessed over three seasons. Furthermore, there was only one year of data to show the effect of seasonal fluctuations on gut bacterial communities of hooded crane. As we did not collect data on dietary resources, we cannot provide direct evidence on the effect of diet on intestinal bacterial community composition of hooded cranes. These limitations should be clarified in future studies.

## Figures and Tables

**Table 1:** Differences in the bacterial community composition based on the similarity test of ANOSIM.

Treatment	ANOSIM	
	*r*	*p*
Fall vs. Winter	0.378	0.001
Fall vs. Spring	0.732	0.001
Winter vs. Spring	0.843	0.001

**Table 2:** SIMPER analysis showing the contribution of bacterial OTU to the differences between seasons. Taxonomic abbreviations: c, class; f, family; g, genus.

OTU	Taxa	Contribution (%)		
		Fall vs. Winter	Fall vs. Spring	Winter vs. Spring
11063	*g__Lactobacillus*	15.80	13.29	15.61
11375	*g__Enterococcus*	12.93	10.80	−
11271	c__Alphaproteobacteria	10.60	−	9.26
11646	c__Mollicutes	8.19	7.02	−
9558	*g__Paenibacillus*	3.43	−	−
5995	f__Enterobacteriaceae	−	14.23	13.60
1494	*g__Pseudomonas*	−	5.87	5.52
6855	f__Enterobacteriaceae	−	−	5.42

## Supplementary Materials

The following are available online at https://www.mdpi.com/2076-2615/10/4/701/s1. Table S1: The distribution of data was analyzed using the Kolmogorov-Smirnov test. Table S2: Sequencing and OTU classification information. Table S3: Relative abundance of the dominant phyla in all samples and the three seasons. Table S4: Relative abundance of the dominant class in all samples and the three seasons. Table S5: Indicator species of the seasons. Table S6: Relative abundance of the dominant genus in all samples and the three seasons. Figure S1: Study area. (A) Shengjin Lake. (B) Western Lindian. Figure S2: Relative abundance of gut bacterial phyla of hooded crane in the three seasons. Sequences that cannot be classified into any known group are assigned as ‘Others’ bacteria.
