# Supplementary material for: Significant Differences in the Gut Bacterial Communities of Hooded Crane (Grus monacha) in Different Seasons at a Stopover Site on the Flyway
Source: Animals (Basel). 2020 Apr 17;10(4):701. doi: 10.3390/ani10040701 (PMC7222709; doi:10.3390/ani10040701)
Supplement: Supplementary file 1 [file animals-10-00701-s001.pdf]

animals

MDPI

Supporting Information

Table S1. The distribution of data was analyzed using the Kolmogorov-Smirnov test.

	Kolmogorov-Smirnov test (*P* value)	Distribution
Bacterial Chao1	0.055	Normal
Bacterial OTU richness	0.312	Normal
Bacterial PD	0.078	Normal
Bacterial shannon	0.959	Normal
Firmicutes	0.052	Normal
Proteobacteria	0.075	Normal
Tenericutes	0.000	Non-normal
Cyanobacteria	0.701	Normal
Actinobacteria	0.546	Normal

Normal distribution: $P > 0.05$; Non-normal distribution: $P < 0.05$.

Table S2. Sequencing and OTU classification information.

Sample	Sequences	OTU	Phylum	Class	Order	Family	Genus
Winter1	28122	1256	20	43	96	167	241
Winter2	33044	1042	23	48	95	179	266
Winter3	39446	1562	24	46	101	177	266
Winter4	35894	1932	28	66	124	213	339
Winter5	38902	2016	29	70	139	239	365
Winter6	38121	1919	23	63	126	218	344
Winter7	34860	862	15	31	63	114	173
Winter8	34429	764	21	41	82	137	186
Winter9	38452	1666	29	70	135	227	343
Winter10	33051	1131	22	52	101	179	262
Winter11	35763	1094	18	37	82	150	229
Winter12	42193	2334	31	84	154	255	407
Winter13	59174	1772	29	70	138	234	352
Winter14	42251	2261	34	89	174	290	448
Winter15	59237	1918	31	75	154	264	416
Winter16	50493	713	16	26	55	107	155
Winter17	34446	1017	20	45	94	169	254
Winter18	33654	1496	26	58	125	222	342
Winter19	32734	2377	34	85	165	272	414
Winter20	34802	2353	30	72	142	248	392
Spring1	27883	423	9	17	39	66	94
Spring2	33610	480	11	21	41	70	98
Spring3	31339	516	14	25	46	85	108
Spring4	49820	356	8	15	26	45	63
Spring5	57106	801	15	27	53	88	134
Spring6	40459	309	8	18	32	49	67
Spring7	57215	1051	15	27	59	100	152
Spring8	36061	1041	13	24	52	91	138
Spring9	34726	1319	18	38	71	122	177
Spring10	32264	446	10	15	31	55	76
Spring11	32773	645	11	21	42	73	115
Spring12	36213	960	12	28	55	100	149
Spring13	32855	499	11	18	35	61	90
Spring14	28725	374	8	13	23	38	67
Spring15	26799	1098	14	29	60	96	151

Spring16	36183	439	9	17	35	58	79
Spring17	29365	799	11	18	45	81	124
Spring18	30234	676	15	25	43	67	102
Spring19	32906	892	15	25	48	83	120
Spring20	29405	461	8	17	34	60	89
Fall1	32467	831	18	50	101	185	268
Fall2	33598	1259	20	47	94	178	275
Fall3	37192	216	10	18	31	63	73
Fall4	29997	612	14	28	49	99	146
Fall5	35037	258	10	17	29	52	64
Fall6	37172	269	14	29	46	81	101
Fall7	35412	823	17	42	83	159	217
Fall8	35909	698	14	26	50	92	133
Fall9	28516	938	12	41	82	158	246
Fall10	57961	156	9	16	27	57	64
Fall11	32239	168	11	17	28	43	56
Fall12	34509	731	13	31	57	116	172
Fall13	30425	391	16	39	69	116	162
Fall14	39234	177	7	13	22	31	33
Fall15	34480	315	13	23	37	64	80
Fall16	34954	443	11	18	42	94	137
Fall17	32646	405	12	21	33	69	93
Fall18	33658	243	14	25	44	73	88
Fall19	34140	219	10	23	40	70	81
Fall20	55857	342	14	22	36	60	75

Summary of sequencing read analysis, numbers of OTUs, and numbers of OTUs that can be classified into different levels (phylum, class, order, family and genus).

Table S3. Relative abundance of the dominant phyla in all samples and the three seasons.

Phylum	Distribuntion	P	Relative abundance (%)		
			Fall	Winter	Spring
Firmicutes	Normal	<0.001	69.4 (28.1)^a	69.7 (22.8)^a	26.4 (21.4)^b
Proteobacteria	Normal	<0.001	10.1 (17.1)^b	23.2 (24.2)^b	72.1 (21.6)^a
Tenericutes	Non-normal	0.003	12.5 (23.2)^a	0.000 (0.001)^b	0.206 (0.852)^{ab}
Cyanobacteria	Normal	0.07	4.33 (9.97)^a	1.45 (2.13)^a	0.016 (0.021)^a
Actinobacteria	Normal	<0.001	1.00 (0.893)^b	3.10 (2.92)^a	0.200 (0.209)^b

The values in brackets represent the standard deviation of the mean. The distribution of data was analyzed by Kolmogorov-Smirnov test. Normal distribution: $P > 0.05$, One way ANOVA. Different letters behind brackets represent significant differences from Tukey HSD comparisons ($P < 0.05$).Non-normal distribution: $P < 0.05$, KW. Different letters behind brackets represent significant differences from Mann-Whitney-Wilcoxon test ($P < 0.05$).

Table S4. Relative abundance of the dominant class in all samples and the three seasons.

Class	Distribution	P	Relative abundance (%)		
			Fall	Winter	Spring
Bacilli	Normal	<0.001	68.8 (28.3)^a	65.3 (21.4)^a	25.9 (20.6)^b
Gammaproteobacteria	Normal	<0.001	8.45 (16.3)^b	4.42 (3.82)^b	67.4 (22.1)^a
Alphaproteobacteria	Normal	<0.001	1.27 (2.62)^b	17.8 (20.9)^a	4.32 (11.2)^b
Mollicutes	Non-normal	0.003	12.5 (23.2)^a	0.000 (0.001)^b	0.206 (0.852)^{ab}
Clostridia	Normal	0.001	0.590 (1.71)^b	4.36 (5.81)^a	0.496 (1.46)^b
Actinobacteria	Normal	<0.001	0.823 (0.732)^b	2.69 (2.70)^a	0.169 (0.198)^b

The values in brackets represent the standard deviation of the mean. The distribution of data was analyzed by Kolmogorov-Smirnov test. Normal distribution: $P > 0.05$, One way ANOVA. Different letters behind brackets represent significant differences from Tukey HSD comparisons ($P <$

0.05).Non-normal distribution: $P < 0.05$, KW. Different letters behind brackets represent significant differences from Mann-Whitney-Wilcoxon test ($P < 0.05$).

Table S5. Indicator species of the seasons.

Group	Indicator species	Taxonomy	P	Relative abundance (%)
Fall	OTU11375	g__*Enterococcus*	0.001	7.495
	OTU11646	c__Mollicutes	0.002	4.292
	OTU2963	g__*Lactobacillus*	0.040	2.969
	OTU8566	s__*ruminis*	0.001	0.840
	OTU8989	s__*ruminis*	0.001	0.260
	OTU4276	g__*Anaerobiospirillum*	0.008	0.248
	OTU10388	g__*Lactobacillus*	0.001	0.220
	OTU10268	g__*Lactobacillus*	0.001	0.199
	OTU1740	g__*Fusobacterium*	0.001	0.140
	OTU11435	g__*Geodermatophilus*	0.001	0.113
	OTU11689	g__*Enterococcus*	0.001	0.104
	OTU8976	g__*Enterococcus*	0.001	0.100
Winter	OTU11063	g__*Lactobacillus*	0.010	19.294
	OTU11271	c__Alphaproteobacteria	0.002	6.967
	OTU9558	g__*Paenibacillus*	0.001	1.961
	OTU10846	g__*Bacillus*	0.010	1.462
	OTU5725	g__*Paenibacillus*	0.001	1.118
	OTU11670	g__*Lactobacillus*	0.001	0.486
	OTU1128	f__Peptostreptococcaceae	0.001	0.332
	OTU11079	g__*Bacillus*	0.005	0.324
	OTU10101	g__*Paenibacillus*	0.001	0.310
	OTU1793	g__*Paenibacillus*	0.001	0.260
	OTU7932	g__*Legionella*	0.015	0.212
	OTU4436	g__*Paenibacillus*	0.001	0.212
	OTU11325	f__Pseudomonadaceae	0.002	0.204
	OTU208	g__*Paenibacillus*	0.001	0.186
	OTU12453	g__*Lactobacillus*	0.007	0.185
	OTU10651	g__*Arthrobacter*	0.001	0.183
	OTU9272	f__Planococcaceae	0.002	0.178
	OTU11343	g__*Solibacillus*	0.001	0.145
	OTU3186	g__*Arthrobacter*	0.001	0.136
	OTU10396	s__*ruminis*	0.001	0.114
	OTU10078	g__*Carnobacterium*	0.001	0.103
Spring	OTU5995	f__Enterobacteriaceae	0.001	9.892
	OTU1494	g__*Pseudomonas*	0.002	4.043
	OTU6855	f__Enterobacteriaceae	0.001	3.504
	OTU11217	g__*Pseudomonas*	0.036	0.800
	OTU5897	f__Enterobacteriaceae	0.001	0.588
	OTU11541	f__Planococcaceae	0.005	0.419
	OTU6251	g__*Pseudomonas*	0.001	0.312
	OTU8048	g__*Pseudomonas*	0.001	0.309
	OTU3626	f__Enterobacteriaceae	0.001	0.307
	OTU8013	g__*Trabulsiella*	0.001	0.251
	OTU5922	g__*Pseudomonas*	0.026	0.241
	OTU10929	f__Enterobacteriaceae	0.001	0.240
	OTU4687	g__*Enterobacter*	0.001	0.204
	OTU10818	g__*Bacillus*	0.021	0.145
	OTU8538	f__Enterobacteriaceae	0.001	0.142
	OTU8921	f__Enterobacteriaceae	0.001	0.125
	OTU6973	f__Enterobacteriaceae	0.001	0.124
	OTU19	g__*Pseudomonas*	0.001	0.117
	OTU4343	f__Enterobacteriaceae	0.001	0.113
	OTU932	g__*Serratia*	0.050	0.112
	OTU11658	g__*Pseudomonas*	0.001	0.101

Taxonomic abbreviations: p, phylum; c, class; o, order; f, family; g, genus; s, species.

Table S6. Relative abundance of the dominant genus in all samples and the three seasons.

Genus	Distribution	P	Relative abundance (%)		
			Fall	Winter	Spring
Lactobacillus	Normal	<0.001	36.5 (27.4)[a]	39.6 (25.4)[a]	3.28 (4.79)[b]
Enterococcus	Normal	<0.001	22.5 (29.2)[a]	0.312 (0.743)[b]	3.11 (3.36)[b]
Pseudomonas	Normal	<0.001	2.24 (4.67)[b]	2.15 (2.44)[b]	18.2 (21.4)[a]
Paenibacillus	Normal	<0.001	0.044 (0.121)[b]	14.8 (18.8)[a]	1.64 (1.87)[b]
Bacillus	Normal	0.063	0.485 (0.890)[a]	4.27 (6.36)[a]	3.52 (6.48)[a]
Solibacillus	Normal	0.092	0.058 (0.111)[a]	1.88 (3.36)[a]	2.00 (4.17)[a]
Turicibacter	Non-normal	<0.001	2.42 (10.5)[b]	0.649 (2.10)[a]	0.079 (0.259)[b]

The values in brackets represent the standard deviation of the mean. The distribution of data was analyzed by Kolmogorov-Smirnov test. Normal distribution: $P > 0.05$, One way ANOVA. Different letters behind brackets represent significant differences from Tukey HSD comparisons ($P < 0.05$).Non-normal distribution: $P < 0.05$, KW. Different letters behind brackets represent significant differences from Mann-Whitney-Wilcoxon test ($P < 0.05$).

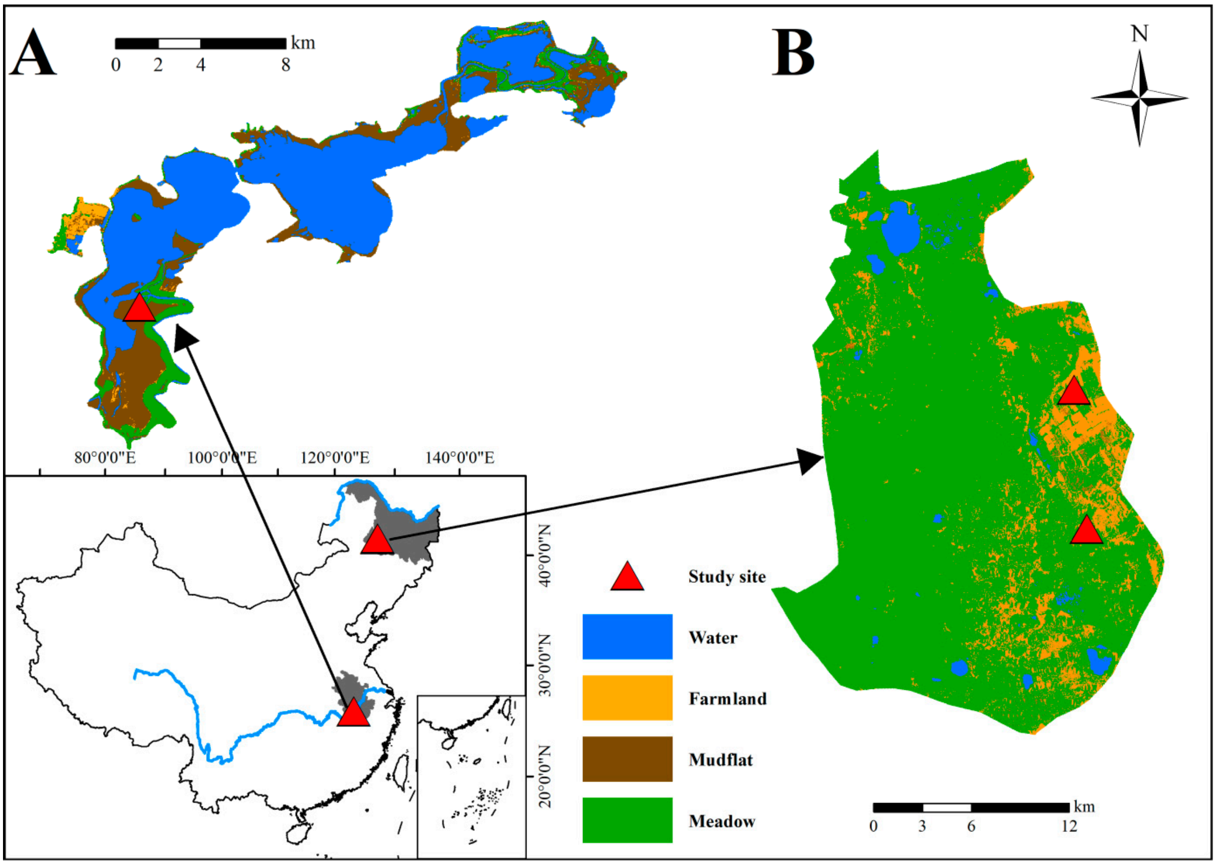

Figure S1. Study area. (A) Shengjin Lake. (B) Western Lindian.

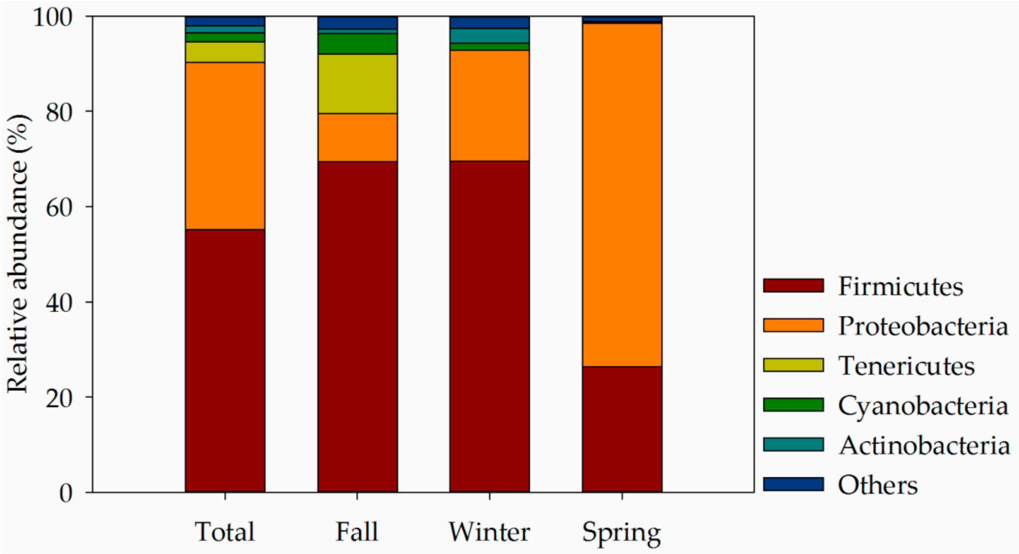

Figure S2. Relative abundance of gut bacterial phyla of hooded crane in the three seasons. Sequences that cannot be classified into any known group are assigned as 'Others' bacteria. .

© 2020 by the authors. Licensee MDPI, Basel, Switzerland. This article is an open access article distributed under the terms and conditions of the Creative Commons Attribution (CC BY) license (http://creativecommons.org/licenses/by/4.0/).
